# Does sexual dimorphism reflect sexual antagonism? Covariation of female fitness with brothers’ sexual traits and their female homologues in neriid flies

**DOI:** 10.1098/rspb.2025.1814

**Published:** 2025-09-24

**Authors:** Ana Caroline Oliveira Vasconcelos, Russell Bonduriansky

**Affiliations:** ^1^ Evolution and Ecology Research Centre and School of Biological, Earth and Environmental Sciences, UNSW Sydney, Sydney, New South Wales, Australia

**Keywords:** intralocus sexual conflict, intersexual genetic correlation, sexually antagonistic coevolution, female body shape, Neriidae, *Telostylinus angusticollis*

## Abstract

Alleles favoured by sexual selection in males can reduce fitness when expressed in females, generating intralocus sexual conflict. It remains unclear whether such conflict is fully resolved by the evolution of sexual dimorphism. If conflict persists, then female fitness might covary negatively with secondary sexual trait expression in male relatives and with sexually homologous trait expression in females themselves. However, because secondary sexual traits often exhibit strong condition dependence, a resource-poor developmental environment could weaken these covariances. We tested these predictions by manipulating larval nutrition of neriid flies (*Telostylinus angusticollis*), generating high (rich diet) and low (poor diet) adult sexual dimorphism in head elongation. Consistent with predictions, in rich-diet families where male head elongation was relatively high, females produced low-viability offspring. Moreover, high female head elongation was associated with delayed oviposition. By contrast, in poor-diet families, we found no evidence of negative covariation between female fitness measures and male head elongation, while female head elongation covaried positively with some measures of female fitness. Our results confirm that sexually dimorphic morphology can reflect sexually antagonistic fitness variation, indicating that intralocus sexual conflict remains unresolved in this species. Our results also suggest that the nutritional environment can modulate the signal of sexual antagonism.

## 1. Introduction

Sexual dimorphism is evidence that the sexes undergo different selection pressures [1]. However, the different reproductive strategies of the sexes, combined with the shared genetic basis of trait expression in males and females, can constrain an optimal sex-specific phenotype, generating intralocus sexual conflict over the alleles controlling trait expression [2]. Theory predicts that the evolution of sexual dimorphism can attenuate intralocus sexual conflict by reducing intersexual genetic correlation and the strength of sexually antagonistic selection [2–4]. Nonetheless, intralocus sexual conflict might persist if the sexes remain displaced from their phenotypic optima [5]. Most of the evidence for intralocus sexual conflict comes from studies showing negative intersexual genetic correlation for fitness, whereby some genotypes enhance fitness in one sex but reduce fitness in the other sex (e.g. [6–11]). Much less is known about the phenotypic manifestations of intralocus sexual conflict (e.g. how the expression of dimorphic, homologous traits in the sexes covaries with fitness in each sex [12]). Such evidence could help reveal whether intralocus sexual conflict is fully resolved by the evolution of sexual dimorphism.

**Figure 1.**
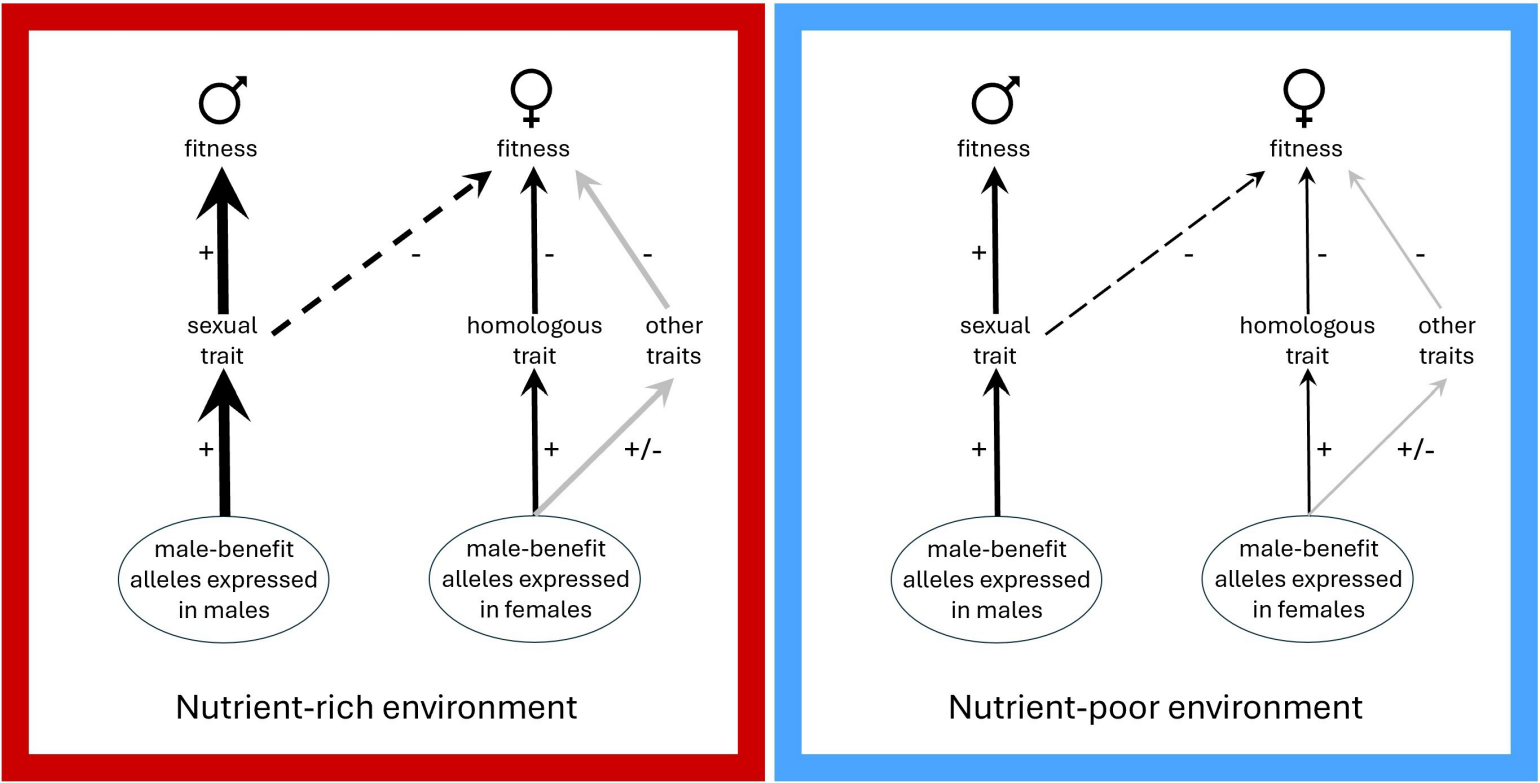
If intralocus sexual conflict remains unresolved, male-benefit alleles that enhance sexually selected morphological traits in males and thereby enhance male fitness (thicker arrows on the left side in each panel, with positive effects indicated by ‘+’) are expected to also enhance the expression of homologous traits in females (thinner arrows on the right side in each panel, with positive effects indicated by ‘+’) and thereby reduce female fitness (negative effect indicated by ‘−’). Pleiotropic effects of such alleles on other female traits (grey arrows) could also reduce female fitness. Such sexually antagonistic fitness variation could manifest as a negative covariance between female fitness and the expression of male relatives’ sexual traits (dashed arrows), as well as the expression of homologous morphological traits in females themselves (solid black arrows). Note that the negative covariance between male trait expression and female fitness here represents a genetic correlation, not direct harm. If the expression of alleles that shape sexually dimorphic morphology is enhanced by dietary nutrients, these covariances could be stronger in a nutrient-rich environment (red/left panel) than in a nutrient-poor environment (blue/right panel).

Traits used as displays or weapons are typically under sexual selection in males, while the homologous traits are not subject to sexual selection in females. However, the shared genetic basis of homologous morphological traits in the sexes (reflected in a positive intersexual genetic correlation for trait expression) might result in the expression in females of traits that confer reproductive benefits for males, such as increased size of sexual weapons or signals [13,14] (figure 1). If intralocus sexual conflict persists, the expression of such male-like phenotypes could impose net costs for females by directly interfering with or diverting resources from survival, foraging or reproduction [15]. Several studies have shown that a correlated response in female fitness is generated when genotypes that confer exaggerated trait expression in males are subjected to artificial selection. For example, in flour beetles, selection for large male mandibles (a secondary sexual trait) resulted in decreased female fertility [16]. Likewise, in bulb mites, selection for enhanced male secondary sexual trait expression resulted in decreased female fitness [17]. Conversely, flour beetle populations subjected to male-specific predation evolved smaller sexually selected mandibles, which indirectly increased female fitness [18]. However, little is known about how female fitness covaries with the expression of secondary sexual traits in male relatives or with the expression of homologous traits in females themselves, particularly within populations that have not been subjected to artificial selection. Quantifying such covariation is important for understanding the role of morphological trait expression in mediating intralocus sexual conflict, as well as revealing the presence of sexually antagonistic variation in natural populations.

In the wild, environmental variation in abundance of resources can generate variation in individual condition—the pool of metabolic resources available to individuals to invest in costly functions [19]. Costly traits, such as secondary sexual traits, often evolve heightened condition dependence because this enables individual optimization of trait expression [19–21]. The evolution of heightened condition dependence might also attenuate intralocus sexual conflict through sex-linked epistatic mechanisms such as hormone signalling [22] that cause metabolic resources to be allocated to secondary sexual traits in males but not in females [15,23]. For example, RNAi-mediated knockdown of the *doublesex* gene in the dung beetle *Digitonthophagus gazella* generated a great reduction in sexual dimorphism in a nutrient-rich environment, suggesting that this gene could act as a mechanism to ameliorate sexual conflict in a context-dependent manner [24]. Nutrient abundance in the environment might therefore affect the covariance between female fitness and male secondary sexual trait expression. The covariance between female fitness and the expression of male secondary sexual traits (and perhaps of the female homologues of such traits, if these traits also exhibit condition-dependent expression) might be elevated in nutrient-rich environments and dampened in nutrient-poor environments because nutrients regulate the expression of genes that shape the development of sexually dimorphic traits [25,26]. Alternatively, if the genetic covariance between the sexes is strengthened under stress or resource limitation, intralocus sexual conflict could be intensified in nutrient-poor environments [27,28]. The role of environment in modulating sexually antagonistic fitness variation remains poorly understood.

**Figure 2.**
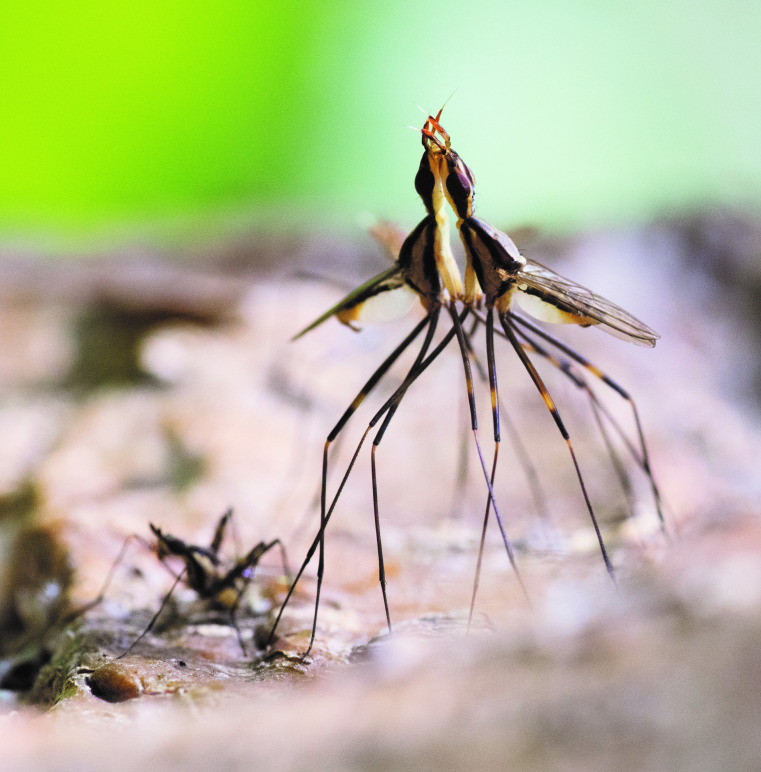
Males of *T. angusticollis* competing over a female ovipositing on tree bark.

**Figure 3.**
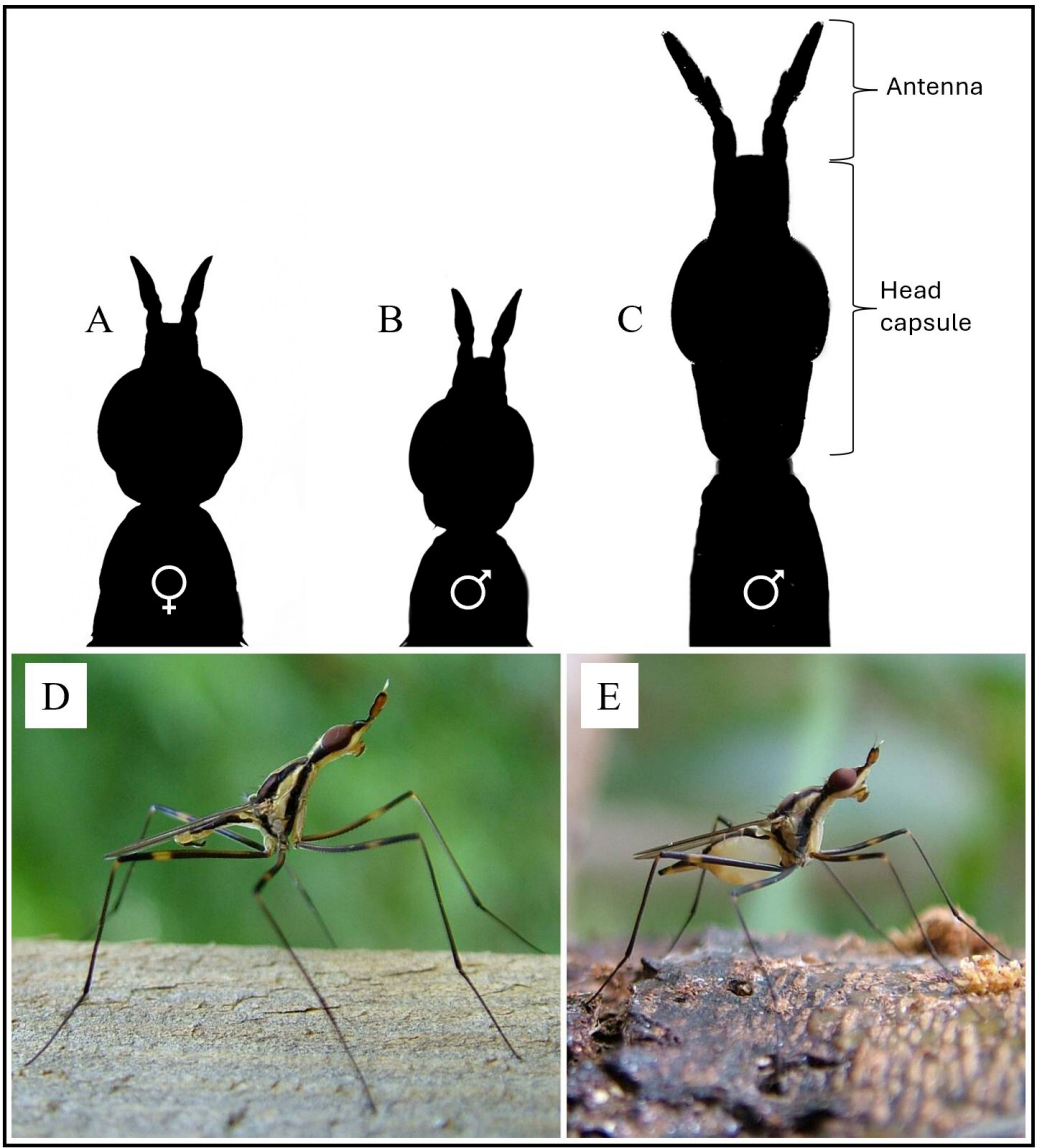
*T. angusticollis* males develop elongated head capsules and antennae when they have access to abundant nutrients as larvae. (A–C) Silhouettes (dorsal view) of *T. angusticollis* female (A) and male (B,C) heads and antennae (antennal arista not shown), illustrating the female-like head shape of males that develop on a nutrient-poor larval diet (B) and the elongated head shape of males that develop on a nutrient-rich larval diet (C). (D) ,E) Photographs of (D) a male and (E) a female viewed from the side.

In the neriid fly *Telostylinus angusticollis*, males have elongated head capsules, antennae and legs, and use these structures as weapons in combat with other males over oviposition sites and females [29–31] (figure 2). The expression of these male secondary sexual traits—especially the length of the head capsule and antennae—is strongly developmentally plastic and condition-dependent [25,32]. Males that are reared on a nutrient-rich larval diet (‘rich diet’) develop highly elongated heads and antennae relative to body size, resulting in pronounced sexual dimorphism in body shape, while males that are reared on a nutrient-poor larval diet (‘poor diet’) exhibit very little elongation of these structures, resulting in a female-like body shape [25,33] (figure 3). *T. angusticollis* females also exhibit somewhat elongated head capsules and antennae (figure 3), but it is not known how the expression of these female homologues of male secondary sexual traits is associated with female fitness.

Because the degree of sexual dimorphism in body shape is strongly affected by larval nutrition in *T. angusticollis*, the expression of male-benefit alleles that affect body shape is likely to be elevated in flies that develop on a rich diet (see [26]). Thus, the covariance between female fitness and the expression of secondary sexual traits in male relatives might be stronger in flies that develop on a rich diet than in those that develop on a poor diet. Likewise, the covariance between female fitness and female body shape might be stronger in a nutrient-rich environment (figure 1). Female fertility often depends strongly on body size in insects [34], but female body shape could also affect female fitness. If a rich larval diet causes elongation of the head capsule and antenna of females as a result of intersexual genetic correlation, the expression of this male-like phenotype could interfere directly with female locomotion or foraging [35–37], and elevated resource investment into these structures could also trade off with investment in reproduction. Moreover, pleiotropic effects of male-benefit alleles on non-homologous female traits could also impose costs for females, strengthening the negative covariance between female fitness and the expression of both male sexual traits and their female homologues.

We used a split-brood design where full siblings from multiple *T. angusticollis* families were randomly allocated to rich and poor larval diets. We then identified families with relatively large and relatively small male secondary sexual traits (combined length of the head capsule and antennae, henceforth ‘head length’) within each larval diet treatment group, and compared reproductive performance (i.e. expression of fitness-related traits), including latency to lay eggs, number of eggs laid and offspring viability, as well as longevity of females in these focal families within each larval diet. If intralocus sexual conflict remains unresolved in this species, then female reproductive performance is predicted to covary negatively with the relative head length of brothers and/or the relative head length of females themselves (figure 1). However, because morphological sexual dimorphism is strongly condition-dependent in this species [25], the signal of intralocus sexual conflict is predicted to be weak or absent in flies reared on poor larval diet. We investigated several female traits because it is not known which traits are most susceptible to intralocus sexual conflict, and we base our conclusions on the overall pattern of results.

## 2. Material and methods

### (a) Experiment set-up

Approximately 30 neriid fly (*T. angusticollis*) individuals were collected from a natural population in Fred Hollows Reserve (33°54′44.04″ S 151°14′52.14″ E), Sydney, Australia, and mixed with flies (around 20 individuals) that had originated from the same location and had been reared in the lab for several generations. The flies were maintained in an 8 l container with a mesh top and layer of cocopeat on the bottom in a controlled-temperature room (approx. 25°C, 12 h light/dark cycle). The flies were supplied with food (sugar and yeast) ad libitum and watered periodically. To collect eggs, we provided oviposition medium in a large Petri dish. The oviposition medium was prepared using rich larval food (described below) that was kept in a room at 25°C for approximately 7 days to allow mould growth and watered and mixed periodically. From this lab stock population, 430 eggs were collected and raised on a standard larval diet in 500 ml containers, with approximately 40 eggs per 200 g of food. The standard larval medium consisted of 10.9 g of soy protein (Nature’s Way brand, Pharm-a-care, Warriewood, NSW, Australia), 29.7 g of brown sugar (Coles brand, Bundaberg, Australia) and 500 ml of water per litre of dry cocopeat. The larval medium was homogenized using a hand-held beater and frozen at −20°C until the day of use. The larval containers were incubated in a controlled-temperature chamber set to 25°C (± 1.5°C) and a 12 h light/dark cycle.

**Figure 4.**
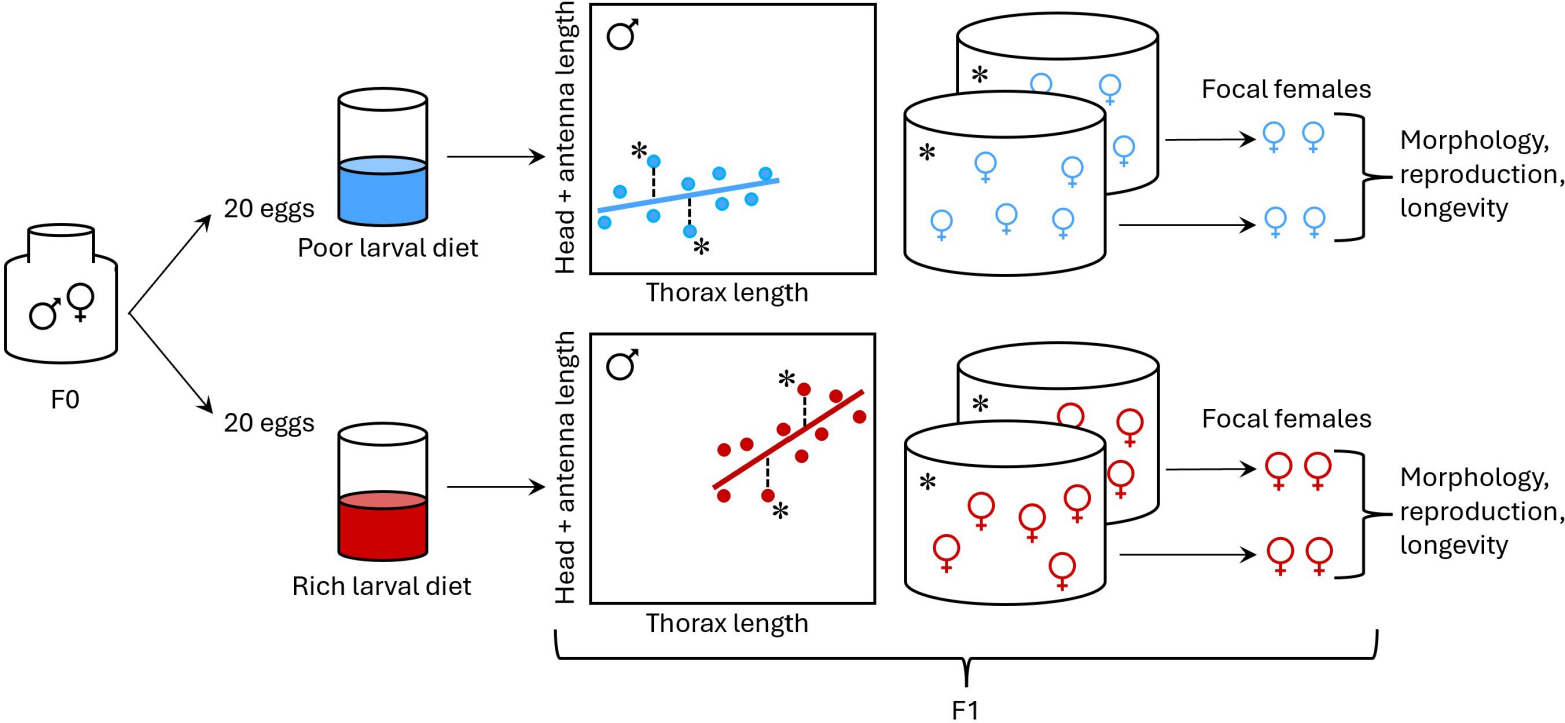
Experimental design: from each of 62 families (F0), eggs were transferred randomly to poor (blue/light) larval diet (20 eggs) and rich (red/dark) larval diet (20 eggs). Two adult brothers (F1) from each family × larval diet combination were imaged and measured, and regression of secondary sexual trait size (‘head length’, calculated as head capsule length + antenna length) on body size (thorax length) was carried out within each larval diet treatment group. Based on this regression, approximately half of the families (those in which the brothers had large mean positive or large mean negative residuals, indicated by an asterisk) were designated as focal families. From each focal family × larval diet combination, two focal females were selected for the fitness assays. Reproductive performance (latency to lay eggs, number of eggs laid, offspring viability) of focal females was assayed by pairing these females with males reared on a standard larval diet. Focal females’ longevity was also recorded. After death, the focal females were imaged and measured to calculate their residual head length.

After the F0 adult flies emerged, 100 male–female pairs were formed and placed in separate 200 ml vials with a substrate of moist cocopeat (figure 4). The pairs were formed randomly, so there is a chance that some pairs consisted of close relatives. However, the number of adult flies in our lab stock population was similar to a typical neriid aggregation (R.B. 2025, personal observation), and the genetic variance of the resulting offspring (F1) population that we used in our experiment should therefore approximate that of naturally occurring neriid populations. After two weeks, when the F0 flies reached maturity [38], a small Petri dish with oviposition medium was provided to each of the pairs, and the flies were given 5 days to oviposit. We collected eggs from pairs that laid at least 40 eggs (*n* = 62 pairs, 2480 eggs in total). From each of these pairs, 20 eggs were transferred to a container with a poor larval diet (20 eggs per 100 g of food), and 20 eggs were transferred to a container with a rich larval diet (20 eggs per 100 g of food). The poor diet consisted of 5.5 g of protein and 14.8 g of brown sugar, whereas the rich diet consisted of 32 g of protein and 89 g of brown sugar, both mixed with 500 ml of water and 1 l of dry cocopeat [25,33]. The vials with the larval medium were placed inside 1 l containers with a layer of cocopeat to facilitate pupation and collection of F1 adult flies (*n* = 1148). The full-sibs descended from an F0 male–female pair and split between rich and poor larval diets constitute a ‘family’ in the analysis. A further 520 eggs were collected from flies that were not used to produce the experimental families and reared on a standard larval diet to generate standard males for crosses used to assess female reproductive performance (see below).

A maximum of 24 h after emergence of the experimental flies (i.e. F1 flies from the 62 families) and standard flies from their puparia, males and females from each family were separated by sex to avoid mating. Upon emergence, two males randomly selected from each family × larval diet combination were allowed 24 h for their exoskeletons to sclerotize fully and then frozen at −20°C for morphological measurements. We chose to measure two males from each family × larval diet combination, as there was little variation in body size or shape within families (family accounted for 91% of total variance in male head length on the poor larval diet and 61% of total variance in male head length on the rich larval diet). Families that did not produce enough males or females were excluded from the analyses. Males were imaged using a Leica MC170HD camera mounted on a Leica MZ16A stereoscope (Wetzlar, Germany). For each male, we measured the length of the thorax as a proxy of body size, and the combined lengths of the head capsule and the right antenna as an index of secondary sexual trait expression, using ImageJ [39]. We used the residuals of the linear regression between family-mean male secondary sexual trait length and family-mean male thorax length to identify the F1 families with low and high expressions of male secondary sexual traits relative to body size within each larval diet treatment group (figure 4). Based on these residuals, we aimed to select approximately half of the families (i.e. approximately corresponding to the upper and lower quartiles for relative head length) as focal families for experimental assays. However, a few families had very uneven sex ratios or could not be used because samples were damaged, and these families were replaced with other families. In total, 12 families from a rich larval diet and 10 families from a poor larval diet with negative residuals and 12 families from a rich larval diet and 10 families from a poor larval diet with positive residuals were chosen for use in the experiment. Of the chosen families, 8 families were in the same quartile on both diets, 24 families were in an upper or lower quartile on one diet only, and 2 families were in opposite quartiles on different diets. Note that, we selected approximately half of the families in each diet treatment group for analysis, and our analysis included a fairly broad range of head elongation values. Because our analysis was done at the family level, we were not able to incorporate variation in body shape between males within families into the analysis.

Two F1 females (replicates) per family × larval diet combination were randomly selected for the reproductive performance and longevity assays. Females were paired with standard males in separate 1 l containers and provided with oviposition medium. Females that developed on a rich diet were paired with males at 10 ± 2 days old, and females that developed on a poor diet were paired with males at 20 ± 2 days old as poor-diet individuals take longer to reach reproductive maturity [38]. Eggs were counted every second day for 8 days. From each female, 20 eggs (where possible) were transferred to containers with a standard diet (20 eggs per 100 g of food) in order to quantify F2 offspring viability, a measure that incorporates egg hatching success and larval and pupal survival until the adult stage. We recorded the number of F2 adults that eclosed from the eggs over 14 days after the first adult eclosion in each replicate brood. To estimate F1 focal female longevity, the focal females were kept with their male partner in the same 1 l containers and provided with food (sugar and yeast) and water but not oviposition medium. Mortality was checked and recorded three times per week. Following death, the focal females were imaged, and their head capsule length, antenna length and thorax length were measured from the images as described above.

### (b) Statistical analyses

We tested whether larval diet manipulation affected male and female body size using a Gaussian linear mixed model with thorax length as the dependent variable, larval diet as the predictor and family identity as a random effect using the package glmmTMB [40]. For this analysis, thorax length was standardized (z-transformed; mean = 0, standard deviation = 1) within sexes but across both larval diet treatments. We used a similar model to test larval diet effects on male and female head length : thorax length ratio (using unstandardized morphological measurements). To test for covariation of relative (residual) head lengths of male and female siblings, we first carried out separate linear regressions of head length on thorax length (both variables standardized within sex × larval diet combinations) within each larval diet × sex combination and obtained the standardized residuals for each individual (representing individual relative head length). We then calculated the standardized family-mean residual for each sex within each larval diet. Finally, we fitted a Gaussian linear mixed model with family-mean female residual head length as the dependent variable and family-mean male residual head length, larval diet and their interaction as fixed effects, as well as family identity as a random effect. We also fitted separate mixed models for each larval diet treatment group. Note that all of the morphological traits that we quantified are strongly positively correlated with body size in this species [25], and using raw trait values would therefore confound covariation of body size and body shape.

We then tested for effects of brothers’ residual head length and focal female relative head length on focal female performance. Theory suggests that sexually antagonistic genetic variation can be maintained at very few loci [41]. The number of sexually antagonistic alleles that might affect brothers’ residual head length and female reproductive performance is thus likely to be small. Hence, in testing for genetic correlation between brothers’ mean residual head length and female reproductive performance, we treated brothers’ mean residual head length as a categorical variable (i.e. positive versus negative family-mean residual male head length within a given larval diet treatment). By contrast, we treated female residual head length as a continuous variable because a female’s own phenotype (reflecting both genetic and environmental variation) could directly affect her fitness. We built linear mixed models that included larval diet (rich versus poor), brothers’ relative head length (positive versus negative residual), individual female relative (i.e. residual) head length, female body size (thorax length, standardized within larval diets to avoid redundancy with the categorical effect of larval diet), and the larval diet × brothers’ residual head length and larval diet × female residual head length interactions as fixed effects using glmmTMB. We used a Gaussian linear mixed model to test effects on the latency to lay eggs and generalized linear mixed models to test effects on the number of eggs laid (Poisson error distribution) and offspring viability (binomial error distribution, eggs that produced viable F2 adults versus eggs that did not produce viable F2 adults). We included an observation-level random effect to account for overdispersion when modelling the number of eggs laid. To test for effects on female survival, we fitted a Cox proportional hazards model using the package coxme [42]. Family identity was included as a random effect in all models. Effects in glmmTMB and coxme models were tested using Wald z-tests. For dependent variables that yielded significant or near-significant interactions, we investigated the data further by carrying out post hoc Tukey tests (for the larval diet × brothers’ residual head length interaction) or separate analyses within each larval diet treatment group (for larval diet × female residual head length interactions). All statistical analyses were conducted using R v. 4.4.2 [43].

## 3. Results

### (a) Effects of larval diet on male and female morphology

**Figure 5.**
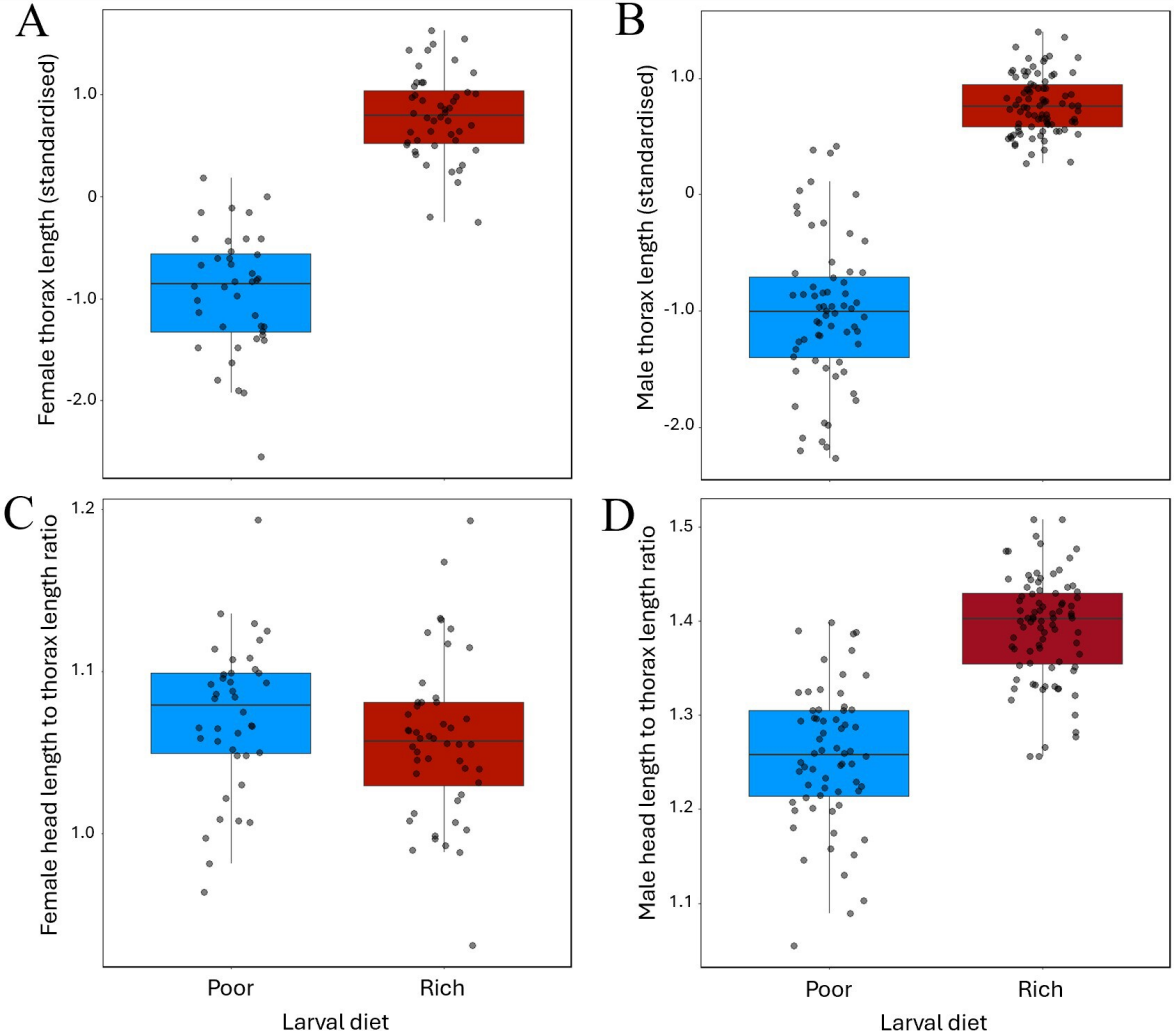
(A,B) Body size (thorax length, standardized) of focal females (A) and their brothers (B) in families reared on rich (dark/red) versus poor (blue/light) larval diets. (C,D) Ration of head length to thorax length of focal females (C) and their brothers (D) in families reared on rich versus poor larval diets. Each point represents an individual male or female. Bars represent medians, boxes show the interquartile range, and whiskers span ±1.5× the interquartile range.

**Figure 6.**
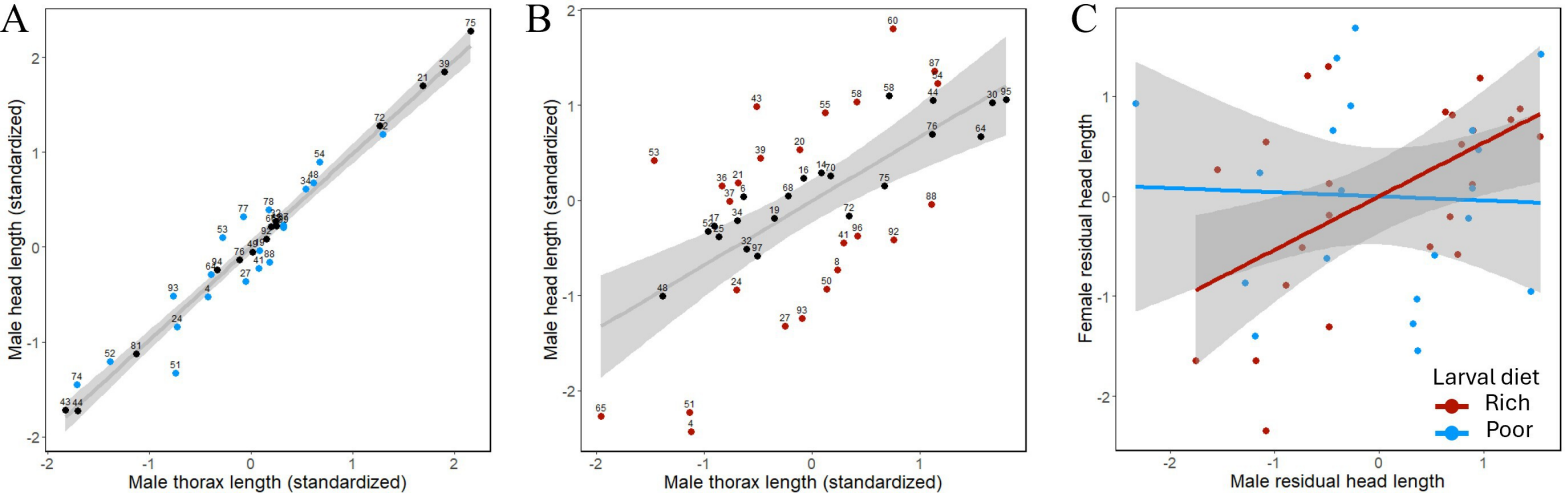
(A,B) Relationship between mean male head length and thorax length among families, shown separately for males reared on rich (A) and poor (B) larval diets. On each larval diet, families chosen as focal families for the experiment are represented by coloured points (blue/light for poor diet, red/dark for rich diet), and non-chosen families are represented by black points. Each point represents the mean of two sibling males (where possible) reared on the same larval diet. Points are labelled by family. (C) Covariation between the family-mean residual head length of focal females and the family-mean residual head length of their brothers within rich and poor larval diets. Families reared on a rich larval diet are shown in red/dark, and families reared on a poor larval diet are shown in blue/light. Residual head length was calculated from within-sex regressions of head length (head capsule length + antenna length) on thorax length. Each point represents a mean value for a focal family × larval diet combination.

We found that larval diet had a strong effect on body size (thorax length) of both sexes (figure 5), with a rich diet increasing mean thorax length relative to a poor diet by 1.79 standard deviations in males (*n* = 148; coeff = 1.758; s.e. = 0.0677; *p* < 0.0001) and 1.73 standard deviations in females (*n* = 88; coeff = 1.702; s.e. = 0.1000; *p* < 0.0001). Larval diet also had a strong effect on the head length : thorax length ratio of males (*n* = 148; coeff = 0.1350; s.e. = 0.0094; *p* < 0.0001), but not of females (*n* = 88; coeff = −0.014; s.e. = 0.0101; *p* = 0.1620), and this sex difference in mean response to larval diet quality resulted in increased morphological sexual dimorphism on the rich larval diet (figure 5). We chose focal families based on the magnitude of the mean male head length residual (figure 6A,B). Among focal families, the relationship between mean residual head length of females and mean residual head length of their brothers differed among larval diet treatments (male residual head length × larval diet interaction: *n* = 44 families; coeff = 0.577, s.e. = 0.278, *p* = 0.0381): we observed a strong, positive relationship among families reared on the rich larval diet (*n* = 24 families; coeff = 0.426, s.e. = 0.143, *p* = 0.0070), but no evidence of a relationship among families reared on the poor larval diet (*n* = 20 families; coeff = −0.071, s.e. = 0.407, *p* = 0.8630; figure 6C).

### (b) Covariation of female performance with their own and their brothers’ morphology

**Figure 7.**
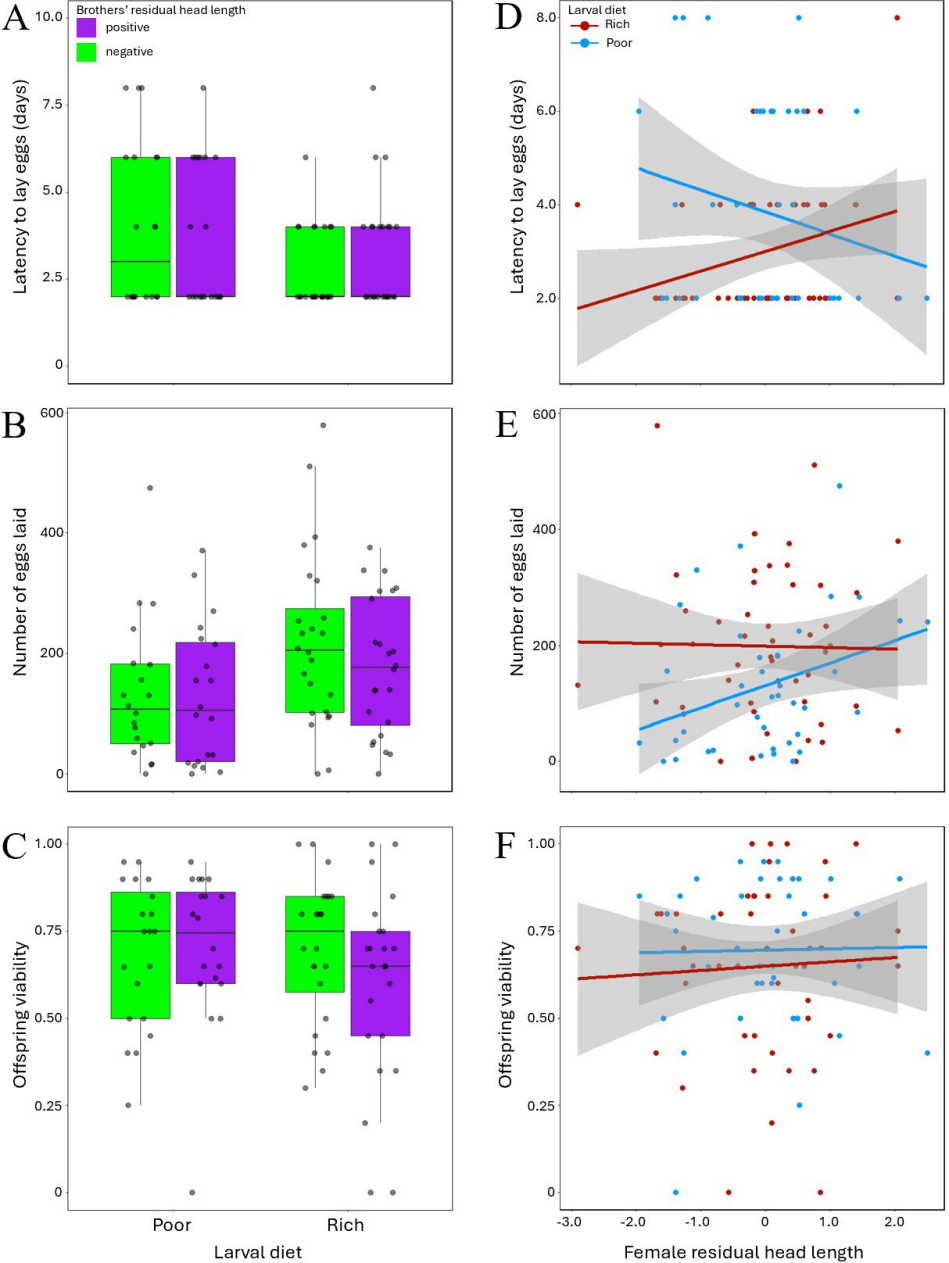
(A–C) Focal females’ latency to lay eggs (A), number of eggs laid (B) and offspring viability (C). Separate boxes are shown for females reared on rich and poor larval diets and, within each larval diet, for families where brothers’ mean residual head length was negative (green/light) versus positive (purple/dark). Bars represent medians, boxes show the interquartile range, and whiskers span ±1.5× the interquartile range. (D–F) Covariation between focal females’ residual head length and latency to lay eggs (D), number of eggs laid (E) and offspring viability (F). Females reared on a rich larval diet are shown in red/dark while females reared on a poor larval diet are shown in blue/light. Residual head length (head capsule length + antenna length) was obtained from regression on thorax length. Each point represents the value for an individual focal female.

Females’ latency to lay eggs did not covary with their brothers’ residual head length (table 1; figure 7A). However, we found evidence that latency covaried with female residual head length, with the larval diet × female residual head length interaction indicating different relationships in rich versus poor larval diet treatments (table 1; figure 7D). Separate tests within larval diet treatments showed a near-significant positive relationship between latency and female residual head length in females reared on the rich larval diet (*n* = 48; coeff = 0.488, s.e. = 0.253, *p* = 0.0540), but a non-significant negative relationship between latency and female residual head length in females reared on the poor larval diet (*n* = 48; coeff = −0.191, s.e. = 0.165, *p* = 0.2460).

**Table 1.** Effects of larval diet treatment, brothers’ residual head length (categorical variable representing the sign of brothers’ mean head length residual), focal female residual head length, focal female thorax length (standardized within larval diets), and the larval diet × brothers’ residual head length and larval diet × female residual head length interactions, on female latency to lay eggs (Gaussian linear mixed model), number of eggs laid (Poisson linear mixed model), offspring viability (binomial linear mixed model) and longevity (Cox proportional hazards model). Family identity was included as a random effect in all models, and an observation-level random effect was included in the model of fecundity to account for overdispersion. The sample size is *n* = 88 focal females for all tests. Effects with *p* < 0.05 are shown in bold.

response variable	effect	coeff	s.e. (coeff)	*z*	*p*-value
latency to lay eggs	intercept	4.322	0.444	9.726	<0.0001
	**larval diet (rich**)	**−1.166**	**0.518**	**−2.250**	**0.0245**
	brothers’ residual head length (positive)	−0.715	0.617	−1.160	0.2459
	female thorax length	−0.051	0.198	−0.260	0.7947
	females’ residual head length	−0.528	0.278	−1.901	0.0573
	larval diet × brothers’ residual head length	0.381	0.866	0.368	0.7131
	**larval diet × female residual head length**	**0.943**	**0.412**	**2.292**	**0.0219**
number of eggs	intercept	4.296	0.280	15.316	<0.0001
	larval diet (rich)	0.685	0.385	1.779	0.0752
	brothers’ residual head length (positive)	−0.071	0.4023	−0.177	0.8596
	female thorax length	−0.134	0.135	−0.994	0.3201
	**females’ residual head length**	**0.506**	**0.204**	**2.476**	**0.0133**
	larval diet × brothers’ residual head length	−0.058	0.560	−0.104	0.9169
	larval diet × female residual head length	−0.507	0.281	−1.802	0.0715
offspring viability	intercept	0.859	0.199	4.305	<0.0001
	larval diet (rich)	0.089	0.195	0.455	0.6492
	brothers’ residual head length (positive)	0.204	0.282	0.725	0.4683
	female thorax length	0.042	0.083	0.502	06159
	females’ residual head length	0.014	0.109	0.131	0.8958
	**larval diet × brothers’ residual head length**	**−0.825**	**0.340**	**−2.430**	**0.0151**
	larval diet × female residual head length	0.015	0.167	0.089	0.9288
longevity	larval diet (rich)	0.091	0.318	0.270	0.7874
	brothers’ residual head length (positive)	0.160	0.325	0.476	0.6944
	female thorax length	0.123	0.125	0.862	0.3887
	females’ residual head length	−0.267	0.174	−1.665	0.0959
	larval diet × brothers’ residual head length	−0.416	0.469	−1.043	0.2969
	larval diet × female residual head length	0.094	0.240	0.441	0.6594

Likewise, females’ fecundity (number of eggs laid) did not covary with their brothers’ residual head length (table 1; figure 7B). However, fecundity covaried with female residual head length and the sign and strength of this relationship appeared to differ between larval diet treatments, although the larval diet × female residual head length interaction was marginally non-significant (table 1; figure 7E). Separate tests within larval diet treatments showed that fecundity did not covary with female residual head length in females reared on the rich larval diet (*n* = 48; coeff = −0.004, s.e. = 0.179, *p* = 0.9800), but covaried positively with female residual head length in females reared on the poor larval diet (*n* = 40; coeff = 0.520, s.e. = 0.222, *p* = 0.0190).

For offspring viability of focal females, we found a larval diet × male residual head length interaction, indicating that offspring viability covaried differently with brothers’ residual head length in the rich versus poor larval diet treatments (table 1; figure 7C). In the rich larval diet treatment group, females that had brothers with positive head length residuals produced less viable offspring than did females that had brothers with negative head length residuals (*n* = 48; Tukey test: coeff = 0.621, s.e. = 0.278, *p* = 0.0258). By contrast, in the poor larval diet treatment group, the viability of offspring produced by focal females did not covary with brothers’ secondary sexual trait size (*n* = 40; Tukey test: coeff = −0.204, s.e. = 0.282, *p* = 0.4683). Offspring viability did not covary with focal females’ residual head length in either larval diet treatment group (table 1; figure 7F).

**Figure 8.**
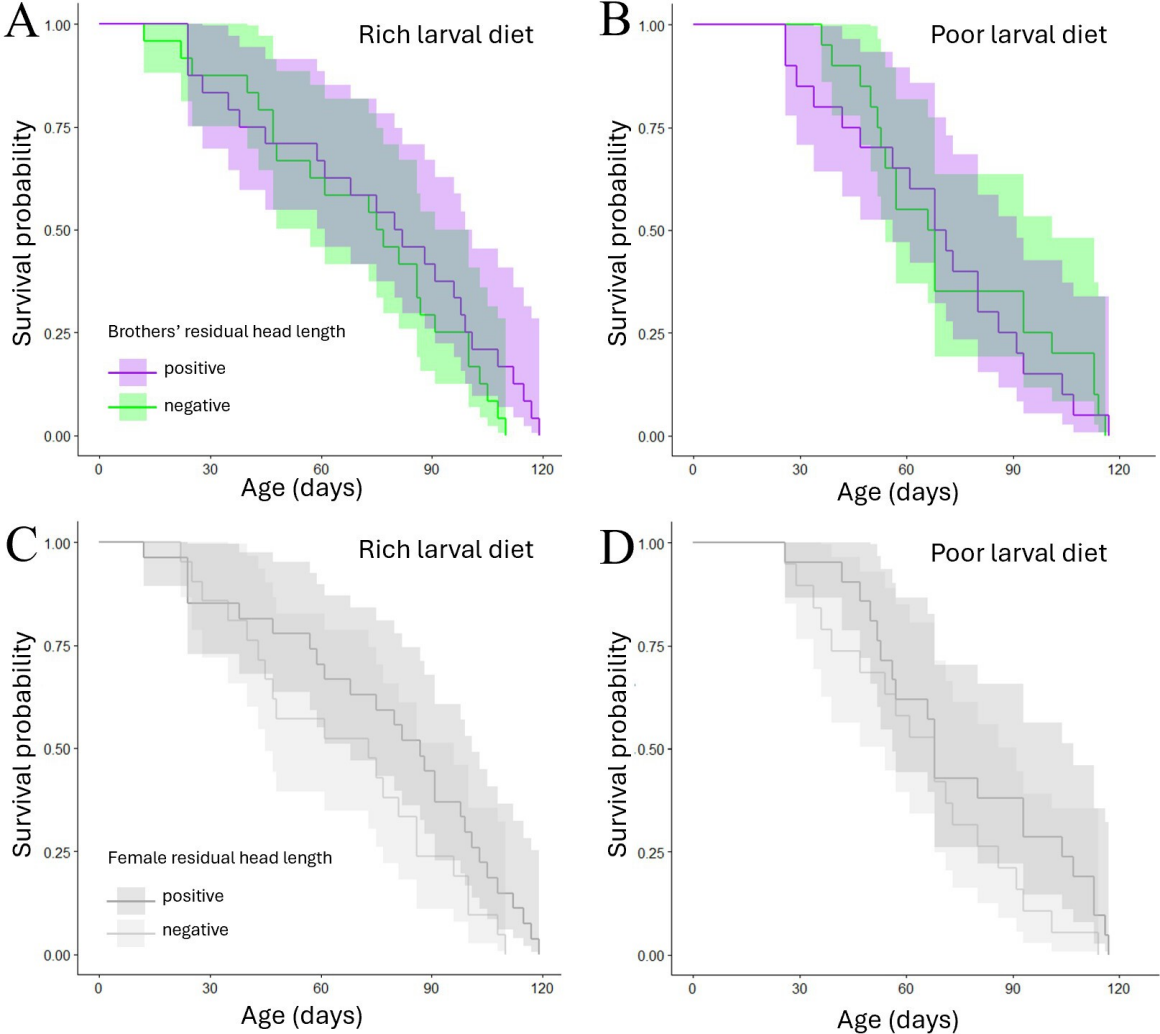
Kaplan–Meier plots representing female survival probability as a function of age. Separate plots are shown for (A) focal females reared on a rich larval diet and whose brothers had positive (purple/dark) versus negative (green/light) residual head lengths, (B) focal females reared on a poor larval diet and whose brothers had positive versus negative residual head lengths, (C) focal females with positive (dark grey) versus negative (light grey) residual head lengths and reared on rich larval diet and (D) focal females with positive versus negative residual head lengths and reared on poor larval diet.

Focal females’ mean longevity was 71 days. We found no evidence that the longevity of focal females covaried with their brothers’ residual head length or their own residual head length in either larval diet treatment group (table 1; figure 8).

## 4. Discussion

If intralocus sexual conflict remains unresolved despite the evolution of sexual dimorphism, then female fitness should covary negatively with the expression of secondary sexual traits in male relatives and with the expression of the sexually homologous traits in females themselves [2,3,44]. Moreover, environmental nutrient abundance could modulate the strength of such covariances by influencing the expression of alleles that affect the expression of sexually dimorphic traits [25,26]. We tested these predictions in *Telostylinus angusticollis*, in which males reared on a rich larval diet express greatly elongated head capsules and antennae (i.e. head length relative to body size), while males reared on a poor larval diet develop female-like morphology [25,33] (figure 3). Consistent with predictions, in flies reared on a nutrient-rich larval diet, we found that families in which males had substantially above-average head elongation also contained females that produced less viable offspring when paired with standard males. Furthermore, increased head elongation in females themselves was associated with longer latency to lay eggs. By contrast, in flies reared on a nutrient-poor larval diet, we found no evidence of negative covariation between female fitness and head elongation of brothers or of females themselves. Instead, increased head elongation in females was associated with reduced latency to lay eggs and higher fecundity. Thus, although results differed among traits, our overall findings suggest that intralocus sexual conflict remains unresolved in *T. angusticollis*, and that sexually antagonistic fitness variation is reflected in the expression of sexually dimorphic morphology. Our results also support the prediction that the strength of the intersexual covariance can be modulated by the developmental environment.

Our results are consistent with the prediction that alleles that cause increased head elongation in *T. angusticollis* males (‘male-benefit alleles’) have detrimental effects on reproductive performance when expressed in females. A potential causal pathway for such effects is increased elongation of the head and antennae in females. Such effects on female body shape might interfere with female foraging or divert resources from reproduction, thereby resulting in direct, negative effects on female performance. Our finding that increased female head elongation was associated with a delay in egg-laying in flies reared on the rich larval diet is consistent with such direct, negative effects of female body shape on female reproduction. Increased latency to lay eggs is likely to be costly because flies experience a high mortality rate in the wild [45–47]. However, this causal pathway does not appear to be sufficient to explain all of our results. Our models included measures of relative head length of both focal females and their brothers. Yet, for offspring viability, we found a negative effect of brothers’ relative head length but no evidence of negative effects of females’ own relative head length. This suggests that the negative effects on offspring viability were mediated by detrimental pleiotropic effects of male-benefit alleles on female traits other than head elongation. Such alleles might have reduced provisioning of eggs, resulting in lower hatching success or reduced offspring viability at the larval or pupal stage. For the number of eggs laid (fecundity), we did not find any effect of either brothers’ relative head length or females’ own relative head length in flies reared on the rich larval diet. Further research is needed to determine why female reproductive traits apparently exhibit varying degrees of sexually antagonistic variation. Overall, for flies reared on the rich larval diet, covariances of female reproductive performance measures with head elongation were either negative or near zero, consistent with intralocus sexual conflict.

By contrast, in flies reared on the poor larval diet, we found that increased head elongation in females was associated with reduced latency to lay eggs and increased fecundity, whereas brothers’ head length did not covary with any female fitness measures. A potential explanation for these findings is that covariation between female head elongation and female fitness reflects the relative effects of sexually antagonistic alleles and condition. The harm to females of expressing male-like traits is expected to depend on the extent to which male-specific selection displaces the female phenotype from the female optimum [2,6,48]. Head and antenna elongation is strongly condition-dependent in males, and, in some genotypes, this trait might be somewhat condition-dependent in females as well [25]. If poor-diet females that have more elongated heads are also in higher condition, condition-dependent expression of reproductive traits might enhance reproductive performance, whereas direct costs of increased head elongation might be too small to negate the positive effects of higher condition. The higher condition of such females could be environmental (e.g. a result of locating a nutrient-rich micro-patch during the larval stage), but it could also have a genetic basis. Indeed, stressful environments (such as our poor-diet treatment) can reveal differences in genetic quality and amplify among-individual variation in performance [27], perhaps including genetic variation at the resource-acquisition loci that are hypothesized to determine the genetic basis of condition [49,50]. The lack of any covariance between brothers’ relative head length and female reproductive performance is also likely to reflect reduced expression of sexually antagonistic alleles in the nutrient-poor environment. Similar to our findings, in seed beetles (*Callosobruchus maculatus*), female fecundity was elevated in isolines in which females expressed male-like colour traits [51]. Overall, for flies reared on the poor larval diet, covariances of female reproductive performance measures with head elongation were either positive or near zero, suggesting weak or less detectable intralocus sexual conflict in this resource-limited environment.

How the environment modulates the expression of sexually antagonistic alleles is still largely unexplored. Sexually antagonistic selection is expected to be stronger in benign (e.g. resource-rich) environments, as the sexes can approach closer to their distinct fitness optima by elevating expression of costly sexually dimorphic traits [26,52]. By contrast, in stressful (e.g. resource-limited) environments, both sexes might be displaced far from their fitness optima, resulting in concordant selection on shared traits [53–55]. However, the available experimental evidence is equivocal and suggests that sexually antagonistic selection can change in intensity and direction depending on the environment and trait measured [27,56–63]. While we only investigated intralocus sexual conflict in this study, environmental nutrient abundance might affect the intensity of interlocus sexual conflict as well: if condition-dependent male sexual traits directly harm females (e.g. during pre-mating struggles), increased nutrient abundance might increase the mean expression of such coercive male traits and the mean level of harm to females [52]. More research is needed to understand how nutrient abundance and other key ecological variables modulate sexually antagonistic fitness variation.

The potential for intralocus sexual conflict to be resolved via the evolution of sex-specific changes in development is still poorly understood [64]. According to theory, if sexual dimorphism matches the optimal phenotype for each sex, intralocus sexual conflict could be fully resolved [2,3,44]. However, empirical evidence suggests that the strength of sexually antagonistic selection is positively correlated with the magnitude of sexual dimorphism, indicating that intralocus sexual conflict often remains unresolved [5]. Even the evolution of sex-limited trait expression might not fully resolve intralocus sexual conflict as a result of pleiotropic effects of sexually antagonistic alleles [65]. The evolution of sex-specific condition dependence could mitigate intralocus sexual conflict but might not resolve the conflict fully, especially if condition dependence itself is subject to a positive intersexual genetic correlation [3,23]. Moreover, variable selection pressures on shared morphological traits could maintain sexually antagonistic alleles via balancing net selection and consequently prevent full conflict resolution [66]. Our results suggest that, in *T. angusticollis*, the evolution of pronounced, condition-dependent sexual dimorphism has not fully resolved intralocus sexual conflict, and that this conflict is manifested in sexually antagonistic variation in the expression of shared morphological traits.

We selected families with the highest and lowest sexual trait expression in males in order to test qualitative predictions. However, this approach complicates the interpretation of parameter values from our models. Our parameter estimates might also be affected by the exclusion of families with highly unequal sex ratios (i.e. families in which males or females failed to emerge) (see [67]). Such families might have had the most strongly sexually antagonistic alleles, resulting in high mortality of embryos or larvae of one sex. We also reared offspring from each family (per diet) in a single vial, which could have resulted in common environment effects on phenotypes. However, common environment effects are unlikely to generate negative covariances between fitness-related traits. Finally, unmeasured morphological, behavioural or life-history traits could also have mediated sexually antagonistic fitness variation in *T. angusticollis* and contributed to our results.

Our study suggests that sexually antagonistic fitness variation can be detected by quantifying the covariation between fitness components and morphological traits of opposite-sex relatives. Few studies have addressed this question thus far (but see [51]). As noted nearly two decades ago by Long & Rice [68], further research is required to understand how morphological, behavioural and life-history traits mediate sexually antagonistic fitness variation and how such variation is modulated by the ambient environment.

## Data Availability

Data and code are available from the Zenodo repository [69].

## References

[1] Darwin C. 1859 On the origin of species by means of natural selection, or the preservation of favoured races in the struggle for life. London, UK: John Murray. PMC5184128 30164232

[2] Lande R. 1980 Sexual dimorphism, sexual selection, and adaptation in polygenic characters. Evolution **34**, 292–305. (10.1111/j.1558-5646.1980.tb04817.x) 28563426

[3] Bonduriansky R, Chenoweth SF. 2009 Intralocus sexual conflict. Trends Ecol. Evol. **24**, 280–288. (10.1016/j.tree.2008.12.005) 19307043

[4] Tosto NM, Beasley ER, Wong BBM, Mank JE, Flanagan SP. 2023 The roles of sexual selection and sexual conflict in shaping patterns of genome and transcriptome variation. Nat. Ecol. Evol. **7**, 981–993. (10.1038/s41559-023-02019-7) 36959239

[5] Cox RM, Calsbeek R. 2009 Sexually antagonistic selection, sexual dimorphism, and the resolution of intralocus sexual conflict. Am. Nat **173**, 176–187. (10.1086/595841) 19138156

[6] Chippindale AK, Gibson JR, Rice WR. 2001 Negative genetic correlation for adult fitness between sexes reveals ontogenetic conflict in Drosophila. Proc. Natl Acad. Sci. USA **98**, 1671–1675. (10.1073/pnas.98.4.1671) 11172009 PMC29315

[7] Fedorka KM, Mousseau TA. 2004 Female mating bias results in conflicting sex-specific offspring fitness. Nature **429**, 65–67. (10.1038/nature02492) 15129280

[8] Foerster K, Coulson T, Sheldon BC, Pemberton JM, Clutton-Brock TH, Kruuk LEB. 2007 Sexually antagonistic genetic variation for fitness in red deer. Nature **447**, 1107–1110. (10.1038/nature05912) 17597758

[9] Mainguy J, Côté SD, Festa-Bianchet M, Coltman DW. 2009 Father–offspring phenotypic correlations suggest intralocus sexual conflict for a fitness-linked trait in a wild sexually dimorphic mammal. Proc. R. Soc. B **276**, 4067–4075. 10.1098/rspb.2009.1231 PMC2825789 19740880

[10] Pischedda A, Chippindale AK. 2006 Intralocus sexual conflict diminishes the benefits of sexual selection. PLoS Biol. **4**, e356. (10.1371/journal.pbio.0040356) 17105343 PMC1618422

[11] Parrett JM, Chmielewski S, Aydogdu E, Łukasiewicz A, Rombauts S, Szubert-Kruszyńska A, Babik W, Konczal M, Radwan J. 2022 Genomic evidence that a sexually selected trait captures genome-wide variation and facilitates the purging of genetic load. Nat. Ecol. Evol. **6**, 1330–1342. (10.1038/s41559-022-01816-w) 35851852

[12] Price PD, Parkus SM, Wright AE. 2023 Recent progress in understanding the genomic architecture of sexual conflict. Curr. Opin. Genet. Dev. **80**, 102047. (10.1016/j.gde.2023.102047) 37163877

[13] Buzatto BA, Tomkins JL, Simmons LW, Machado G. 2014 Correlated evolution of sexual dimorphism and male dimorphism in a clade of neotropical harvestmen. Evolution **68**, 1671–1686. (10.1111/evo.12395) 24593685

[14] Clark CJ, Rankin D. 2020 Subtle, pervasive genetic correlation between the sexes in the evolution of dimorphic hummingbird tail ornaments. Evolution **74**, 528–543. (10.1111/evo.13881) 31729031

[15] Bonduriansky R, Rowe L. 2005 Sexual selection, genetic architecture, and the condition dependence of body shape in the sexually dimorphic fly Prochyliza xanthostoma (Piophilidae). Evolution **59**, 138–151. (10.1111/j.0014-3820.2005.tb00901.x) 15792234

[16] Harano T, Okada K, Nakayama S, Miyatake T, Hosken DJ. 2010 Intralocus sexual conflict unresolved by sex-limited trait expression. Curr. Biol. **20**, 2036–2039. (10.1016/j.cub.2010.10.023) 21055943

[17] Plesnar Bielak A, Skrzynecka AM, Miler K, Radwan J. 2014 Selection for alternative male reproductive tactics alters intralocus sexual conflict. Evolution **68**, 2137–2144. (10.1111/evo.12409) 24641007

[18] Okada K, Katsuki M, Sharma MD, Kiyose K, Seko T, Okada Y, Wilson AJ, Hosken DJ. 2021 Natural selection increases female fitness by reversing the exaggeration of a male sexually selected trait. Nat. Commun. **12**, 3420. (10.1038/s41467-021-23804-7) 34103535 PMC8187464

[19] Andersson M. 1982 Sexual selection, natural selection and quality advertisement. Biol J Linn Soc **17**, 375–393.

[20] Cotton S, Fowler K, Pomiankowski A. 2004 Do sexual ornaments demonstrate heightened condition-dependent expression as predicted by the handicap hypothesis? Proc. R. Soc. B **271**, 771–783. (10.1098/rspb.2004.2688) PMC1691662 15255094

[21] McAlpine DK. 1979 Agonistic behavior in *Achias australis* (Diptera, Platystomatidae) and the significance of eyestalks. In Sexual selection and reproductive competition in insects (eds MS Blum, NA Blum), pp. 221–230. New York, NY: Academic Press. (10.1016/B978-0-12-108750-0.50012-4)

[22] Gotoh H, Miyakawa H, Ishikawa A, Ishikawa Y, Sugime Y, Emlen DJ, Lavine LC, Miura T. 2014 Developmental link between sex and nutrition; doublesex regulates sex-specific mandible growth via juvenile hormone signaling in stag beetles. PLoS Genet. **10**, e1004098. (10.1371/journal.pgen.1004098) 24453990 PMC3894178

[23] Bonduriansky R. 2007 The genetic architecture of sexual dimorphism: the potential roles of genomic imprinting and condition-dependence. In Sex, size and gender roles (eds DJ Fairbairn, WU Blanckenhorn, T Székely), pp. 176–184. Oxford, UK: Oxford University Press. (10.1093/acprof:oso/9780199208784.003.0020)

[24] Rohner PT, Linz DM, Moczek AP. 2021 Doublesex mediates species-, sex-, environment- and trait-specific exaggeration of size and shape. Proc. R. Soc. B **288**, 20210241. (10.1098/rspb.2021.0241) PMC8220263 34157867

[25] Bonduriansky R. 2007 The evolution of condition-dependent sexual dimorphism. Am. Nat. **169**, 9–19. (10.1086/510214) 17206580

[26] Wyman MJ, Agrawal AF, Rowe L. 2010 Condition-dependence of the sexually dimorphic transcriptome in Drosophila melanogaster. Evolution **64**, 1836–1848. (10.1111/j.1558-5646.2009.00938.x) 20059540

[27] Berger D, Grieshop K, Lind MI, Goenaga J, Maklakov AA, Arnqvist G. 2014 Intralocus sexual conflict and environmental stress. Evolution **68**, 2184–2196. (10.1111/evo.12439) 24766035

[28] Long TAF, Agrawal AF, Rowe L. 2012 The effect of sexual selection on offspring fitness depends on the nature of genetic variation. Curr. Biol. **22**, 204–208. (10.1016/j.cub.2011.12.020) 22226747

[29] Fricke C, Adler MI, Brooks RC, Bonduriansky R. 2015 The complexity of male reproductive success: effects of nutrition, morphology, and experience. Behav. Ecol. **26**, 617–624. (10.1093/beheco/aru240)

[30] Hooper AK, Spagopoulou F, Wylde Z, Maklakov AA, Bonduriansky R. 2017 Ontogenetic timing as a condition‐dependent life history trait: High‐condition males develop quickly, peak early, and age fast. Evolution **71**, 671–685. (10.1111/evo.13172) 28067402

[31] Hubakk K, Wylde Z, Bonduriansky R. 2024 Can developmental plasticity shape sexual competition and promote reproductive isolation? Behav. Ecol. **35**, arae047. (10.1093/beheco/arae047)

[32] Bonduriansky R, Head M. 2007 Maternal and paternal condition effects on offspring phenotype in Telostylinus angusticollis (Diptera: Neriidae). J. Evol. Biol. **20**, 2379–2388. (10.1111/j.1420-9101.2007.01419.x) 17956399

[33] Sentinella AT, Crean AJ, Bonduriansky R. 2013 Dietary protein mediates a trade‐off between larval survival and the development of male secondary sexual traits. Funct. Ecol. **27**, 1134–1144. (10.1111/1365-2435.12104)

[34] Blanckenhorn WU. 2000 The evolution of body size: what keeps organisms small? Q. Rev. Biol. **75**, 385–407. (10.1086/393620) 11125698

[35] Emlen DJ. 2001 Costs and the diversification of exaggerated animal structures. Science **291**, 1534–1536. (10.1126/science.1056607) 11222856

[36] McCullough EL, Weingarden PR, Emlen DJ. 2012 Costs of elaborate weapons in a rhinoceros beetle: how difficult is it to fly with a big horn? Behav. Ecol. **23**, 1042–1048. (10.1093/beheco/ars069)

[37] Nijhout HF, Emlen DJ. 1998 Competition among body parts in the development and evolution of insect morphology. Proc. Natl Acad. Sci. USA **95**, 3685–3689. (10.1073/pnas.95.7.3685) 9520426 PMC19896

[38] Wylde Z, Spagopoulou F, Hooper AK, Maklakov AA, Bonduriansky R. 2019 Parental breeding age effects on descendants’ longevity interact over 2 generations in matrilines and patrilines. PLoS Biol. **17**, e3000556. (10.1371/journal.pbio.3000556) 31765371 PMC6901263

[39] Schneider CA, Rasband WS, Eliceiri KW. 2012 NIH image to imageJ: 25 years of image analysis. Nat. Methods **9**, 671–675. (10.1038/nmeth.2089) 22930834 PMC5554542

[40] McGillycuddy M, Warton DI, Popovic G, Bolker BM. 2025 Parsimoniously fitting large multivariate random effects in glmmTMB. J. Stat. Softw. **112**, 1–19. (10.18637/jss.v112.i01)

[41] Flintham E, Savolainen V, Otto SP, Reuter M, Mullon C. 2025 The maintenance of genetic polymorphism underlying sexually antagonistic traits. Evol. Lett. **9**, 259–272. (10.1093/evlett/qrae059) 40191410 PMC11968185

[42] Therneau T. 2010 Coxme: mixed effects cox models. Vienna, Austria: R Foundation for Statistical Computing.

[43] R Core Team. 2021 R: a language and environment for statistical computing. Vienna, Austria: R Foundation for Statistical Computing.

[44] Arnqvist G, Rowe L. 2005 Sexual conflict. Princeton, NJ: Princeton University Press.

[45] Bonduriansky R, Brassil CE. 2002 Rapid and costly ageing in wild male flies. Nature **420**, 377–377. (10.1038/420377a) 12459773

[46] Kawasaki N, Brassil CE, Brooks RC, Bonduriansky R. 2008 Environmental effects on the expression of life span and aging: an extreme contrast between wild and captive cohorts of Telostylinus angusticollis (Diptera: Neriidae). Am. Nat. **172**, 346–357. 18710341 10.1086/589519

[47] Mautz BS, Rode NO, Bonduriansky R, Rundle HD. 2019 Comparing ageing and the effects of diet supplementation in wild vs. captive antler flies, Protopiophila litigata. J. Anim. Ecol. **88**, 1913–1924. (10.1111/1365-2656.13079) 31368156

[48] Connallon T, Cox RM, Calsbeek R. 2010 Fitness consequences of sex-specific selection. Evolution **64**, 1671–1682. (10.1111/j.1558-5646.2009.00934.x) 20050912

[49] Rowe L, Houle D. 1996 The lek paradox and the capture of genetic variance by condition dependent traits. Proc. R. Soc. B **263**, 1415–1421. (10.1098/rspb.1996.0207)

[50] Houle D. 1991 Genetic covariance of fitness correlates: what genetic correlations are made of and why it matters. Evolution **45**, 630–648. (10.1111/j.1558-5646.1991.tb04334.x) 28568816

[51] Berger D, Martinossi-Allibert I, Grieshop K, Lind MI, Maklakov AA, Arnqvist G. 2016 Intralocus sexual conflict and the tragedy of the commons in seed beetles. Am. Nat. **188**, E98–E112. (10.1086/687963) 27622882

[52] Flintham EO, Savolainen V, Mullon C. 2023 Male harm offsets the demographic benefits of good genes. Proc. Natl Acad. Sci. USA **120**, e2211668120. (10.1073/pnas.2211668120) 36862690 PMC10013744

[53] Connallon T, Hall MD. 2016 Genetic correlations and sex‐specific adaptation in changing environments. Evolution **70**, 2186–2198. (10.1111/evo.13025) 27477129

[54] De Lisle SP, Goedert D, Reedy AM, Svensson EI. 2018 Climatic factors and species range position predict sexually antagonistic selection across taxa. Phil. Trans. R. Soc. B **373**, 20170415. (10.1098/rstb.2017.0415) 30150216 PMC6125731

[55] Plesnar‐Bielak A, Łukasiewicz A. 2021 Sexual conflict in a changing environment. Biol. Rev. **96**, 1854–1867. (10.1111/brv.12728) 33960630 PMC8518779

[56] Delcourt M, Blows MW, Rundle HD. 2009 Sexually antagonistic genetic variance for fitness in an ancestral and a novel environment. Proc. R. Soc. B **276**, 2009–2014. 10.1098/rspb.2008.1459 PMC2677236 19324806

[57] Delph LF, Andicoechea J, Steven JC, Herlihy CR, Scarpino SV, Bell DL. 2011 Environment-dependent intralocus sexual conflict in a dioecious plant. New Phytol. **192**, 542–552. (10.1111/j.1469-8137.2011.03811.x) 21726233

[58] García‐Roa R, Chirinos V, Carazo P. 2019 The ecology of sexual conflict: temperature variation in the social environment can drastically modulate male harm to females. Funct. Ecol. **33**, 681–692. (10.1111/1365-2435.13275)

[59] Koch EL, Sbilordo SH, Guillaume F. 2020 Genetic variance in fitness and its cross‐sex covariance predict adaptation during experimental evolution. Evolution **74**, 2725–2740. (10.1111/evo.14119) 33135158

[60] Londoño-Nieto C, García-Roa R, Garcia-Co C, González P, Carazo P. 2023 Thermal phenotypic plasticity of pre- and post-copulatory male harm buffers sexual conflict in wild Drosophila melanogaster. eLife **12**, e84759. (10.7554/elife.84759) 37102499 PMC10191624

[61] Łukasiewicz A. 2020 Juvenile diet quality and intensity of sexual conflict in the mite Sancassania berlesei. BMC Evol. Biol. **20**, 35. (10.1186/s12862-020-1599-5) 32164531 PMC7069193

[62] Punzalan D, Delcourt M, Rundle HD. 2014 Comparing the intersex genetic correlation for fitness across novel environments in the fruit fly, Drosophila serrata. Heredity **112**, 143–148. (10.1038/hdy.2013.85) 24045292 PMC3907099

[63] Skwierzyńska AM, Radwan J, Plesnar-Bielak A. 2018 Male-limited secondary sexual trait interacts with environment in determining female fitness. Evolution **72**, 1716–1722. (10.1111/evo.13551) 29984827 PMC6175437

[64] Pennell TM, Mank JE, Alonzo SH, Hosken DJ. 2024 On the resolution of sexual conflict over shared traits. Proc. R. Soc. B **291**, 20240438. (10.1098/rspb.2024.0438) PMC11289733 39082243

[65] Rayner JG, Pascoal S, Bailey NW. 2019 Release from intralocus sexual conflict? Evolved loss of a male sexual trait demasculinizes female gene expression. Proc. R. Soc. B **286**, 20190497. 10.1098/rspb.2019.0497 PMC6501929 31014218

[66] Pennell TM, Morrow EH. 2013 Two sexes, one genome: the evolutionary dynamics of intralocus sexual conflict. Ecol. Evol. **3**, 1819–1834. (10.1002/ece3.540) 23789088 PMC3686212

[67] Katsuki M, Harano T, Miyatake T, Okada K, Hosken DJ. 2012 Intralocus sexual conflict and offspring sex ratio. Ecol. Lett. **15**, 193–197. (10.1111/j.1461-0248.2011.01725.x) 22225600

[68] Long TAF, Rice WR. 2007 Adult locomotory activity mediates intralocus sexual conflict in a laboratory-adapted population of Drosophila melanogaster. Proc. R. Soc. B **274**, 3105–3112. 10.1098/rspb.2007.1140 PMC2293944 17925279

[69] Vasconcelos ACO, Bonduriansky R. 2025 Data for: does sexual dimorphism reflect sexual antagonism? Covariation of female fitness with brothers’ sexual traits and their female homologues in neriid flies. Zenodo. (10.5281/zenodo.16888108)40987322

